# Effectiveness of a home-based computerized cognitive training in Parkinson's disease: a pilot randomized cross-over study

**DOI:** 10.3389/fpsyg.2024.1531688

**Published:** 2025-01-09

**Authors:** Serena Tagliente, Brigida Minafra, Simona Aresta, Paola Santacesaria, Andrea Buccoliero, Cinzia Palmirotta, Gianvito Lagravinese, Davide Mongelli, Christian Gelao, Luigi Macchitella, Stefania Pazzi, Domenico Scrutinio, Paola Baiardi, Petronilla Battista

**Affiliations:** ^1^Istituti Clinici Scientifici Maugeri IRCCS, Laboratory of Neuropsychology of Bari Institute, Bari, Italy; ^2^Istituti Clinici Scientifici Maugeri IRCCS, Neurorehabilitation Unit of Bari Institute, Bari, Italy; ^3^University School for Advanced Studies IUSS Pavia, Pavia, Italy; ^4^R&D Department GPI SpA, Università degli Studi di Verona, Verona, Italy; ^5^Unit for Severe Disabilities in Developmental Age and Young Adults (Developmental Neurology and Neurorehabilitation), Associazione “La Nostra Famiglia” - IRCCS “E. Medea”, Scientific Hospital for Neurorehabilitation, Brindisi, Italy; ^6^Consorzio di Bioingegneria e Informatica Medica (CBIM), Pavia, Italy; ^7^Istituti Clinici Scientifici Maugeri IRCCS, Direzione Scientifica Centrale of Pavia Institute, Pavia, Italy

**Keywords:** rehabilitation, telerehabilitation, cognitive training, neuropsychology, PD

## Abstract

**Introduction:**

Cognitive symptoms are common in Parkinson's Disease (PD), and digital interventions like telerehabilitation other an accessible way to manage these symptoms. This study aimed to assess the effectiveness of a Home-Based Computerized Cognitive Training (HB-CCT) program in individuals with PD using a pilot randomized cross-over design.

**Methods:**

Twenty-five participants (mean age 69.32 ± 7.21 years, mean MDS-UPDRS III 33.76 ± 14.25) with PD and mild cognitive impairment were enrolled. They underwent neuropsychological assessments at three time points (5-week intervals): Baseline, after the HB-CCTi, and after Standard Care. The HB-CCT consisted of the Neurotablet^®^ platform that was used to target cognitive domains such as Attention, Memory, Perception, Executive Functioning and Language. All participants completed both the Neurotablet intervention and Standard Care blocks in a randomized order. After a Shapiro-Wilk test, non-parametric repeated measures analyses of variance (Friedman's test) and *post-hoc* comparisons corrected with the Benjamini-Hochberg approach were performed to compare the effects on primary and secondary cognitive outcomes over experimental intervention and Standard Care.

**Results:**

The results from the Friedman analysis revealed significant improvements in Word List Immediate Recall, Digit Span Forward and Complex Figure Recall (all *p* < 0.001) following the HB-CCT, compared to the Baseline. Additionally, Naming performance showed significant improvement after the HB-CCT (*p* = 0.02). Significant differences were also observed when comparing the HB-CCT with Standard Care, with improved performance in TMT-A (*p* = 0.02), Phonemic Fluency (*p* < 0.01), and Digit Span Forward (*p* < 0.01).

**Discussion:**

These findings suggest that HB-CCT via Neurotablet can effectively enhance specific cognitive abilities in PD, supporting the role of digital, home-based interventions as feasible strategies to mitigate cognitive decline.

## Introduction

Parkinson's Disease (PD) is a progressive neurodegenerative disorder caused by a prominent loss of dopaminergic neurons in the substantia nigra pars compacta with the result of dopamine deficiency within the basal ganglia structures. The presence in the substantia nigra of aggregates of α-synuclein, known as Lewy bodies, is the neuropathological hallmark of the disease (Kalia et al., 2015). This process leads to a variety of motor symptoms (bradykinesia, muscular rigidity, rest tremor, and postural and gait impairment) (Buchman et al., 2012). However, PD is also associated with numerous non-motor symptoms (hyposmia, sleep disorders, depression, constipation), some of which precede the motor dysfunction (Schapira et al., 2017).

Among non-motor symptoms, cognitive changes are frequently observed, even in the initial phases of the disease, with Mild Cognitive Impairment (MCI) affecting around 30–40% of individuals (Cosgrove et al., 2015). These cognitive changes can affect a person's independence and have a significant clinical impact, as it is related to institutionalization, mortality, and increased caregiver burden (Watson and Leverenz, 2010). Longitudinal studies have shown that approximately 50% of individuals with PD develop dementia after 10 years. Cognitive deficits may impact a person's autonomy and affect adherence to treatment due to an inability to understand the effects of medication or to follow a prescribed regimen (Bainbridge and Ruscin, 2009).

Impairment in different cognitive domains has been described in individuals with PD, affecting primarily attention and executive functions, memory, and visuo-spatial functioning (Verbaan et al., 2007; Aarsland et al., 2021; Wallace et al., 2022). Among attentional disorders, Allcock et al. (2009) showed that vigilance and reaction time, together with attentional fluctuations, are more frequently reported. Furthermore, several subcomponents of executive functioning, such as verbal fluency, planning, problem-solving, working memory, and set-shifting, are impaired in individuals with PD, reflecting frontostriatal damage. In addition, patients exhibit deficits in working memory, long-term memory, and learning.

Interventions that target neuropsychological deficits could play a crucial role in enhancing overall quality of life. Several studies showed that individuals with PD may benefit from the verbal cue, suggesting that the new information is recorded but not readily accessible and that amnesia is mainly due to executive dysfunction (Emre, 2003), rather than a real dysfunction of the hippocampal structures. Moreover, visuo-spatial impairment involves both visuo-perceptual and visuo-motor abilities independently of cognitive decline (Girotti et al., 1988).

To date, pharmacological therapies have been the only available treatments that provide symptom relief. While pharmacological therapies are crucial in managing PD, significant limitations underscore the necessity for continued research and development of alternative treatment modalities. The current pharmacological treatments primarily alleviate motor symptoms, such as tremors, rigidity, and bradykinesia. However, they do not modify the disease's progression or significantly improve cognitive symptoms (Antonini et al., 2021). Among cholinesterase inhibitors, rivastigmine is an approved treatment for Parkinson's dementia and has demonstrated efficacy in improving cognitive and neuropsychiatric symptoms (Reingold et al., 2007; Mamikonyan et al., 2015; Oh et al., 2015). Pharmacological therapies addressing cognitive symptoms, such as cholinesterase inhibitors (e.g., rivastigmine and donepezil), have been associated with adverse drug reactions (Sun and Armstrong, 2021). Moreover, it should be noted that the response to pharmacological interventions can vary considerably between individuals with PD. Factors such as disease stage, severity, and the presence of comorbid conditions can influence the efficacy of treatment. Some of them may eventually require surgical interventions like deep brain stimulation when pharmacological treatments become less effective. However, not all patients are suitable candidates for surgery (Minafra et al., 2014; Servello et al., 2023). As of now, no treatment has been demonstrated to halt or reverse the underlying neurodegenerative process. Consequently, non-pharmacological interventions that address the neuropsychological difficulties associated with PD may be pivotal in enhancing the overall quality of life of people living with this pathology (Sun and Armstrong, 2021).

Regarding specific interventions on cognitive functions, there is considerable inconsistency in the terminology used in the literature regarding cognitive stimulation, cognitive training, and cognitive rehabilitation for people presenting cognitive impairment. In particular, cognitive training and rehabilitation are often used interchangeably despite coming from different disciplines and having different objectives (Clare et al., 2003; Paggetti et al., 2024; Pinto et al., 2024). Cognitive rehabilitation aims to identify functional goals relevant to the person living with cognitive impairment and work toward achieving them with the support of family members and/or caregivers. The emphasis is on improving or maintaining functioning in daily life, building on the person's strengths, and finding ways to compensate and/or sustain independence. Cognitive rehabilitation does not focus on improving cognitive function, but addresses disability resulting from the impact of cognitive impairment on daily functioning and activities. Cognitive stimulation includes a series of activities and discussions (usually in a group) that aim to improve general cognitive and social functioning. Cognitive training uses guided practice on standardized paper and pencil or computerized cognitive tasks, with adaptable intensity and difficulty. It is based on a series of specific exercises and tasks aimed at improving single or multiple cognitive functions, and can be performed individually or in group sessions (Clare et al., 2003; Gavelin et al., 2020).

Despite the structural brain changes associated with the progression of neurodegenerative processes, cognitive training in PD has been shown to significantly increase functional brain connectivity and activation. This intervention resulted in improvements in cognition and functional disability, with long-term effects maintained for up to 18 months (Díez-Cirarda et al., 2018; Gavelin et al., 2022; Giustiniani et al., 2022). Growing evidence supports the benefits of cognitive intervention, yet individuals with PD still encounter many barriers to accessing rehabilitation services. Therefore their referral is suboptimal, likely due to skepticism regarding the value of intervention in the context of neurodegeneration (Battista et al., 2023; Pinto et al., 2024), the scarcity of sources in the healthcare care system of many countries (Balikuddembe and Reinhardt, 2020; Suárez-González et al., 2024), the lack of awareness regarding the role of the neuropsychological rehabilitation amongst referrers, and the geographical barriers that impede access to in-person cognitive rehabilitation services (Zaman et al., 2021). Furthermore, intensive and prolonged periods of training are emerging as crucial for chronic conditions, making it difficult to afford for all individuals (Vellata et al., 2021). These barriers may be mitigated by capitalizing upon alternative intervention modalities, such as telerehabilitation, an application of telemedicine that concerns the remote delivery of a variety of rehabilitative services through telecommunication technology (Piron et al., 2009), which has shown promising in treating individuals with PD (Vellata et al., 2021; Maggio et al., 2024).

Home-based teleneuropsychology enables individuals with comorbidities or motor disabilities to engage in cognitive training from home, enhancing their abilities and maintaining mental function through accessible, remote technology. Telerehabilitation offers flexibility in scheduling and is often more cost-effective in terms of time and money. Compared to smartphones, tablet-based tools for teleneuropsychology are particularly accessible for older adults, thanks to user-friendly screens and clearly defined response areas. These platforms provide real-time feedback and automatic adjustments to match users' skill levels (Hammers et al., 2020; Naamanka et al., 2024). This approach places patients at the center of their rehabilitation, allowing them to view progress charts that objectively reflect their improvements. Continuous contact between patient and clinician ensures that the clinician's guidance remains a key factor in successful training outcomes. Notably, telerehabilitation has also been associated with patients' subjective perceptions of cognitive, emotional, and physical improvements (Mosca et al., 2020). Several studies have also demonstrated the effectiveness of telerehabilitation treatments based on video games and virtual reality (Herz et al., 2013; Maggio et al., 2018). These methods have shown comparable effectiveness to face-to-face therapy in improving motor and non-motor symptoms and quality of life of individuals with PD (Cacciante et al., 2022). The integration of physical and cognitive functions stimulate the brain's reward system, increasing motivation and program adherence. In addition, telerehabilitation enables a larger group of individuals to work on a task at once, with fewer medical staff needed, and allows the clinician to monitor the progress in real-time (Vellata et al., 2021). It reduces time and costs, and allows even daily intensive exercise while keeping the person in his social and physical environment (McCue et al., 2010).

For the present study, we compared a new Home-Based Computerized Cognitive Training (HB-CCT) with Standard Care in PD patients, implementing a cross-over design. The experimental HB-CCT intervention was delivered with a platform named Neurotablet^®^, with the aim of evaluating its potential effectiveness in enhancing cognitive performance in individuals with PD.

## Materials and methods

### Study design

**Figure 1:**
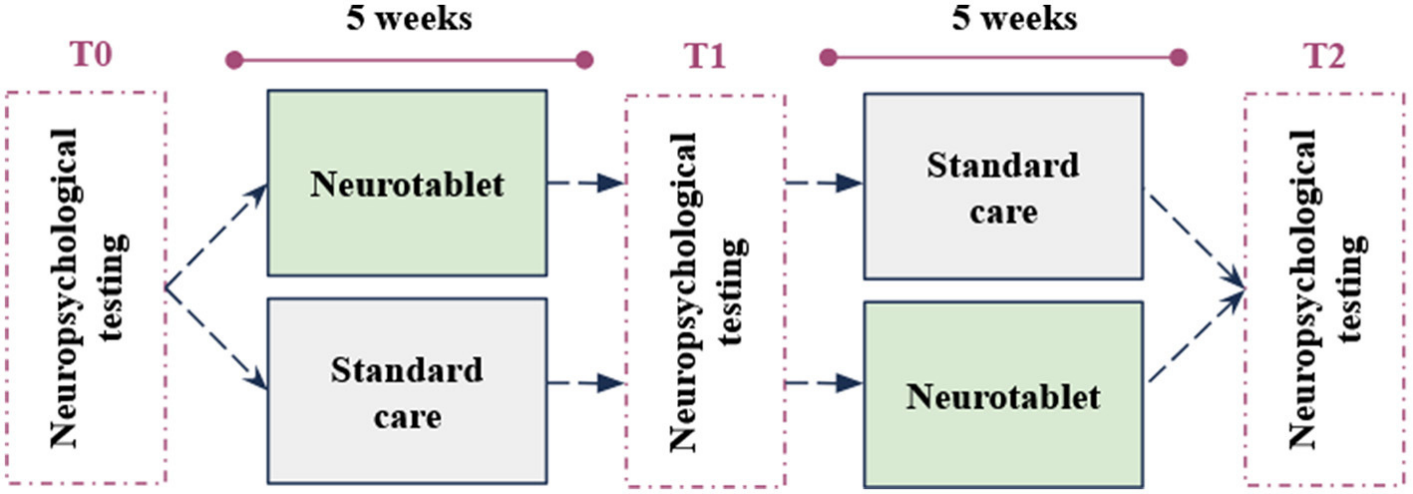
The procedure of the pilot cross-over randomized study design.

We conducted a pilot cross-over randomized repeated measures study including two groups, with three testing time points (T0-T1-T2) at 5-week intervals (Figure 1). All participants underwent a neuropsychological assessment (T0) administered by expert neuropsychologists. After the first neuropsychological assessment (T0), participants were blindly allocated to Group 1 undergoing the experimental intervention or to Group 2 undergoing Standard Care. After 5 weeks, Group 1 and Group 2 patients returned to the Laboratory and underwent a second neuropsychological assessment (T1). At this point, the conditions for both groups changed. While Group 1 took Standard Care at home, Group 2 practiced the experimental intervention for 5 weeks. Finally, at the end of this period, all participants came again to the Hospital for the last neuropsychological examination (T2).

### Participants

Individuals who met the current clinical criteria for a PD diagnosis according to the UK Parkinson's Disease Society Brain Bank (Hughes et al., 1992) and presented with MCI diagnosed according to the Movement Disorders Society (MDS) criteria (Litvan et al., 2012) were enrolled in this study upon providing their written informed consent. Other inclusion criteria were age between 40 and 85 years, at least 5 years of education (Primary School), and Hoehn and Yahr (H&Y) stage < 3. The exclusion criteria encompassed the presence of other neurological or psychiatric disorders, the presence of dementia as measured by the Montreal Cognitive Assessment (MoCA < 15.50) (Santangelo et al., 2015). The cognitive screening of participants was conducted using the Montreal Cognitive Assessment (MoCA) (Nasreddine et al., 2005), which is renowned for its reliable psychometric properties and is widely recommended for identifying Mild Cognitive Impairment (MCI) in Parkinson's Disease (Gill et al., 2008; Hoops et al., 2009; Nazem et al., 2009; Dalrymple-Alford et al., 2010; Kandiah et al., 2014). The MoCA has been validated in the Italian population, yet there remains no consensus on cut-off scores for detecting MCI or dementia in Italy.

In our study, we based our cut-offs on the work of Santangelo et al. (2015) as our participants were recruited from Southern Italy, which closely aligns with their sample population. While other normative studies from Northern Italy provide valuable insights, we felt that their cut-offs might not be as representative of our specific population.

Following Santangelo et al.'s criteria, we categorized participants as follows: (i) MoCA < 15.5 indicating severe impairment (major cognitive deficits), (ii) 15.5 ≤ MoCA < 17.54 suggesting the presence of MCI, and (iii) MoCA ≥ 17.54 denoting normal cognitive status.

Further exclusion criteria were: a history of alcohol or drug abuse, undergoing a concomitant cognitive training treatment during the study period, changes in drug therapy during the study period, and the presence of uncorrected visual or auditory disturbances that may limit the administration of the test and/or treatment.

All of them completed the pilot randomized cross-over study design. The study protocol was approved by the Institutional Review Board of the IRCCS Giovanni Paolo II Hospital (No. Prot. 1195). Participants were recruited between March 2023 and March 2024 from the Laboratory of Neuropsychology at the Clinical Scientific Institutes Maugeri of Bari, Italy. All participants were Italian speakers and functionally monolingual. Demographic data, including age, sex, and years of education, along with clinical data, including the disease duration, the levodopa (l-dopa) equivalent daily dosage (LEDD), the H&Y (Hoehn and Yahr, 2011) and the MDS-Unified Parkinson's Disease Rating Scale (MDS-UPDRS–part III), were collected from patients during the “ON” phase.

**Figure 2:**
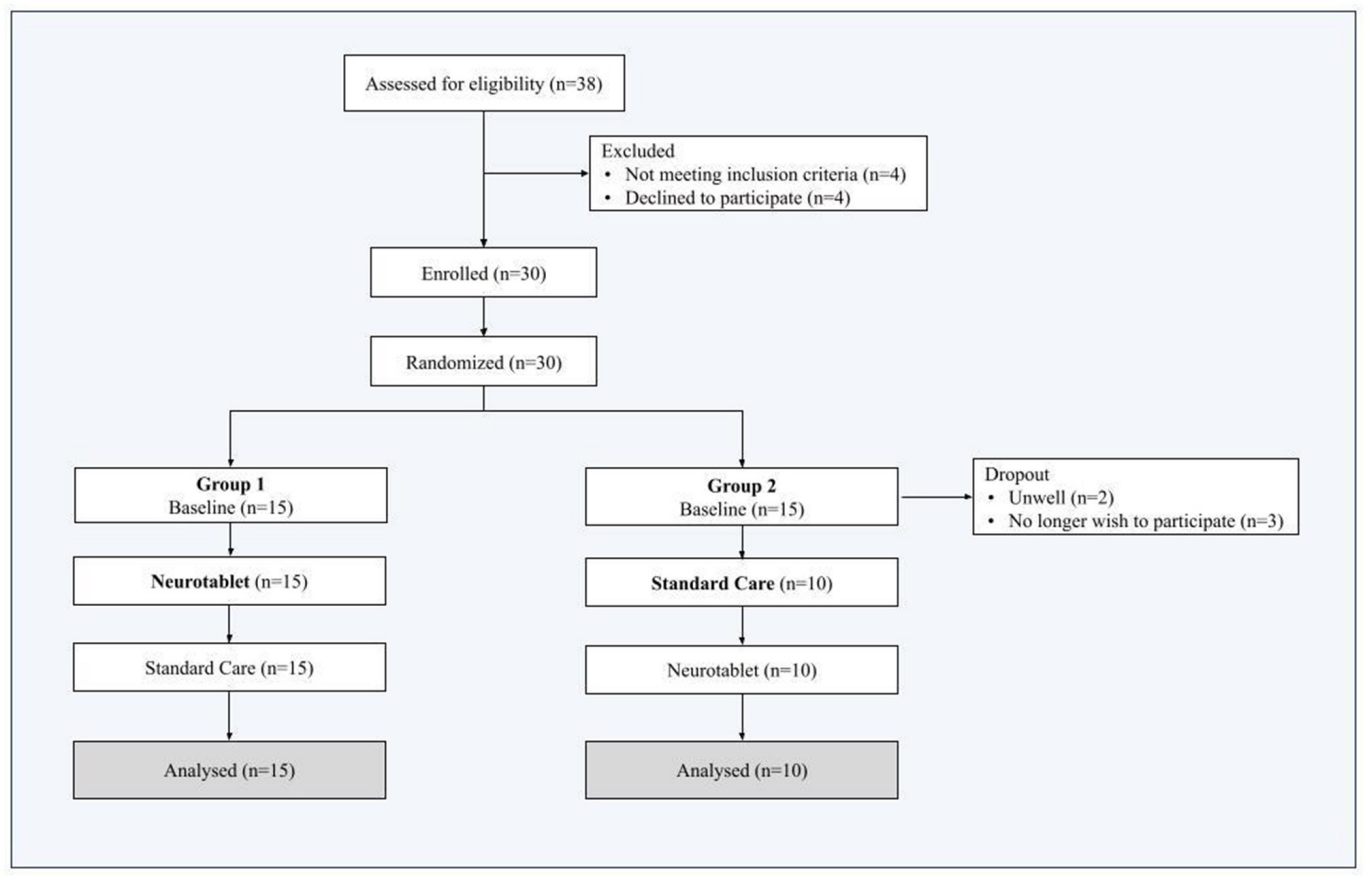
Attrition details of the enrollment process.

Thirty-eight individuals were initially assessed for eligibility. Eight out of 38 were excluded because they did not meet the participant inclusion criteria (*n* = 4) or declined to participate (*n* = 4). Thus, thirty subjects were randomized in Group 1 and Group 2 and completed the Baseline. We then registered 5 more dropouts from Group 2, as *n* = 2 participants were unwell and *n* = 3 participants decided no longer to participate in this study. The final Group 2 sample included *n* = 10 participants, while the final Group 1 sample included *n* = 15 participants. Therefore, the final sample included twenty-five participants, who performed both Neurotablet and Standard Care (attrition details are outlined in Figure 2).

Participants had a mean age of 69.32 ± 7.21 years (range 55–85) and a mean education of 13.00 ± 4.51 years. Additional information regarding demographic and clinical data is reported in Supplementary Table S1.

### Randomization and blinding

A researcher blinded to participants' identities and not involved in enrollment or testing used a randomization minimization procedure (Altman and Bland, 2005) to allocate participants to therapy first (Group 1) or Standard Care (Group 2). To mitigate potential assessor bias, outcome assessors were also blinded. To minimize potential differences between groups we took into account the severity of cognitive impairment (MoCA scores) at Baseline.

### Intervention and standard care blocks

The intervention block consisted of 5 weeks of daily Home-Based Computerized Cognitive Training (HB-CCT), namely Neurotablet, with a target dose of 45 min/day and 2 tele-therapy sessions per week of 45 min to monitor patients' progression. The Standard Care block consisted of regular health advice.

To take part in this study, all participants were provided with a web-based platform called Neurotablet, which was installed on a Samsung Galaxy tablet, as well as a stable internet connection in order to enable their participation in teletherapy sessions. Participants were instructed to register and log into a personal profile uploaded on the website platform. Participants were assisted with software installation and were provided with a document containing written instructions and screenshots of each step of the installation process. If issues arose with the installation process, participants were asked to access reliable assistance from someone who could provide support, as needed, during testing and treatment sessions. Additional assistance was provided, as needed, either via phone or by using TeamViewer© (http://www.teamviewer.com), which allowed short-term remote access to the participant's computer.

The Neurotablet is a sophisticated multi-platform system for cognitive rehabilitation, combining robust hardware and software to deliver a seamless therapeutic experience. The device features a tablet equipped with a capacitive touchscreen, ensuring precision in user interactions. Its connectivity suite includes Wi-Fi and Bluetooth, enabling secure and efficient data transfer for remote monitoring and programmability. The software framework is a core of the Neurotablet, powered by a custom-configured operating system optimized for neurorehabilitation applications. Neurotablet offers a library of over 40 different modular exercises and an amount of 10,000 customizable difficult levels. Each level incorporates predefined thresholds to ensure gradual progression, fostering sustained engagement and challenge, when participants reach the fixed thresholds, the exercise increases in difficulty.

Exercises were classified according to the following cognitive domains: attention, Memory, Perception, Executive Functioning, Language, and Neglect. The exercises employed in this study were specifically tailored to the cognitive profile exhibited by the participants at the neuropsychological examination. The level of difficulty was adapted for each patient through a proprietary adaptive algorithm that dynamically adjusts the difficulty of exercises based on real-time user performance. Key adjustable parameters include the number of stimuli, color distractors, and trial numbers for each session. The interface is designed with gamification elements, ensuring a visually engaging and motivating experience while maintaining accessibility for users with motor impairments. Performance tracking and analytics are integral components, with detailed metrics collected on error rates, task completion times, and adherence levels. The system provides real-time feedback to users during sessions and generates comprehensive progress reports for clinicians. These insights enable informed adjustments to therapy plans, maximizing the intervention's effectiveness. Remote accessibility is facilitated through a secure, cloud-based system that allows clinicians to update therapy modules, monitor progress, and modify task parameters as needed. The device also supports remote programmability, enabling clinicians to customize and modify weekly therapy plans through a secure online interface. These plans adjust exercises to target specific cognitive weaknesses, as evidenced by detailed user performance analytics. The platform's built-in monitoring tools provide precise metrics like error rates, reaction times, and task adherence, enhancing the clinician's ability to track patient progress remotely.

Furthermore, the Neurotablet incorporates advanced algorithmic features, including an automated shut-down after 5 min of inactivity to prevent unproductive sessions and ensures compliance with rehabilitation goals. The intervention model emphasizes user engagement through visually interactive elements akin to gamification, fostering motivation and long-term participation. The Neurotablet is built with a strong emphasis on security and compliance, adhering to GDPR and HIPAA standards to ensure the confidentiality and integrity of patient data. Further details about the platform used for the intervention are reported in Supplementary material.

### Outcome measures

The primary outcomes of the neuropsychological testing were measures of memory, attention, and executive functions. Specifically, we used the following tests to assess memory: Digit Span Forward (Monaco et al., 2015), Rey Auditory Verbal Learning Test (RAVLT) Immediate and Recall (Carlesimo et al., 1996), and the Rey–Osterrieth Complex Figure (ROCF) Recall (Caffarra et al., 2002b). Attention and executive functions were assessed by Digit Span Backward (Monaco et al., 2015), Trail Making Test (TMT A-B; Giovagnoli et al., 1996), Stroop test–Brief version (Caffarra et al., 2002a) and Phonemic fluency test (Carlesimo et al., 1996).

The secondary outcome measures included additional cognitive tests that assessed general cognitive efficiency, as well as visuo-constructive, executive and linguistic abilities: MoCA, the Clock Drawing Test (CDT); (Caffarra et al., 2011), Rey–Osterrieth Complex Figure (ROCF) Copy (Caffarra et al., 2002b), Category fluency test (Novelli et al., 1986) and the Screening for Aphasia in NeuroDegeneration (SAND; Catricalà et al., 2017; Battista et al., 2018).

To decrease possible learning effects, we used parallel versions of the MoCA (8.1, 8.2, 8.3). However, not all of the aforementioned cognitive tests have their respective parallel versions, therefore to minimize the learning effect we administered the Repeatable Battery for the Assessment of Neuropsychological Status (RBANS; Randolph et al., 1998) forms A and B at T1 and T2. RBANS is a brief neuropsychological testing battery comprised of 12 subtests used to calculate five index scores: Immediate Memory Index (comprised of List Learning and Story Memory subtests), Visuo-spatial/Constructional Index (Figure Copy and Line Orientation subtests), Attention Index (Digit Span and Coding subtests), Language Index (Picture Naming and Semantic Fluency subtests), and Delayed Memory Index (List Recall, List Recognition, Story Recall, and Figure Recall subtests), and a Total scale score (comprised of all 12 subtests). Higher scores indicate better performance, both for subtests and index scores.

Supplementary Table S2 provides a comprehensive overview of the tests employed in this study, describing each outcome measure.

### Statistical analysis

The sociodemographic and clinical data of the enrolled patients are presented using descriptive statistics. Continuous variables are expressed as mean ± standard deviation (SD), while categorical variables as proportion (%). Neuropsychological measures are presented as median and interquartile range (IQR).

In order to ensure an accurate comparison of performance scores from disparate neuropsychological tests and batteries (including cognitive tests and the RBANS), a min-max normalization was performed. Each test may have a distinct range of scores, varying scales, and differing SD, which can lead to skewed comparisons and potentially misleading interpretations of the data. Min-max normalization is a data preprocessing technique that transforms the original score values into a standardized range, typically between 0 and 1. This approach allowed us to convert scores from different tests to a common scale. This process ensures that all test results are standardized in terms of their range, allowing for direct comparisons. For example, if one test has a maximum score of 30 and another has a maximum score of 80, normalized scores can be expressed equally within the 0 to 1 range, making them directly comparable. Moreover, compared to the raw scores, the absolute numbers might bias analysis, as some tests may have inherently higher or lower score ranges. Normalizing the scores removes this bias, allowing the focus to be on the relative performance across tests rather than on the absolute scores. By applying min-max normalization, we prepared the data ensuring that no single test disproportionately influences the outcome. Normalized scores are also easier to interpret, as they can be considered as proportions of performance. This interpretation can help clinicians understand how an individual's scores compare to the maximum potential score of each test, providing insight into performance levels. Therefore, considering the specific domains, the following tests with different scoring ranges have been scaled: (1) Memory domain: Digit Span Forward (range: 3–9) and RBANS Digit Span (range: 0–16), RAVLT Immediate (range: 0–75) and RBANS List Learning (range: 0–40), RAVLT Recall (range: 0–15) and RBANS List Recall (range: 0–10), ROCF–Recall (range: 0–36) and RBANS Figure Recall (range: 0–20); (2) Visuo-constructive domain: ROCF–Copy (range: 0–36) and RBANS Figure Copy (range: 0–20); (3) Language domain: Naming SAND (range: 0–14) and RBANS Naming (range: 0–10).

Following the normalization and merging of selected tests and their parallel forms, the subsequent statistical analyses were conducted. To this end, tests grouped according to their respective cognitive domains have been hereinafter designated as follows: Digit Span Forward, Word List Immediate, Word List Recall, Complex Figure Recall, Complex Figure Copy, Naming. Tests not referenced herein were not subjected to standardization, and the original nomenclature has been utilized.

The analysis involved repeated measures on the same individuals at three distinct time points: at Baseline, after HB-CCT Neurotablet intervention and after Standard Care. The neuropsychological outcomes at each assessment were treated as between-groups factors, where the groups were represented by the sample at Baseline, the sample after the experimental intervention (Neurotablet) and the sample after 5 weeks of Standard Care (Standard Care). A non-parametric one-way repeated measures analysis of variance, i.e., Friedman's test has been performed, to compare Baseline, Neurotablet and Standard Care. The output of the Friedman's test indicates whether there are statistically significant differences in the scores across the assessments, independently from the order of intervention delivery. *Post-hoc* comparisons were corrected with the Benjamini-Hochberg correction. If significant differences are found, *post hoc* analyses can determine specifically which pairs of comparisons (e.g., Baseline vs. Neurotablet, Neurotablet vs. Standard Care, Baseline vs. Standard Care) are driving those differences. Kendall's W effect size (ES) and the 95% confidence intervals (CI) measures were computed between conditions.

To account for inter-individual variability we also implemented the Linear Mixed-Effects models (LMM). These models included *Conditions* as *fixed effect* and a *random intercept* for *each participant* to account for within-individual correlations, using Restricted Maximum Likelihood for parameter estimation. The model was fitted using the *lmer* function in the *lme4* package in R.

The significance level adopted was 5% (*p* < 0.05), with 95% confidence intervals. Data were analyzed using the R Studio program version 2024.04.2.

## Results

Patients were randomized to receive either the HB-CCT followed by the Standard Care (Group 1: *n* = 15, age: 67.53 ± 7.26, 5 females, education: 13.53 ± 4.66, disease duration: 6.73 ± 6.15, LEDD: 456.53 ± 184.62, H&Y: 2.37 ± 1.09, MDS-UPDRS III: 32.27 ± 15.02), or Standard Care followed by HB-CCT (Group 2: *n* = 10, age: 72.00 ± 6.58, 2 females, education: 12.20 ± 4.39, disease duration: 12.50 ± 9.73, LEDD: 720.00 ± 303.02, H&Y: 2.75 ± 0.95, MDS-UPDRS III: 36.00 ± 13.47). There were no significant differences between Group 1 and Group 2 for age, education, H&Y severity scale, and MoCA scores at Baseline, in line with the minimization randomization method (*p* > 0.05). Further details are reported in Supplementary Table S3.

Results from the Friedman's test on neuropsychological primary and secondary outcomes at Baseline, after the Neurotablet training and after Standard Care are displayed in Table 1. Furthermore, the LMM results, for each neuropsychological score with fixed and random effects are shown in Table 2.

**Table 1:** Descriptive and Friedman's test analysis of neuropsychological outcomes between the three assessments: at baseline, after the Neurotablet training and after Standard Care.

	**Baseline**	**Neurotablet**	**Standard care**					
**Variables**	**Median (IQR)**	**Median (IQR)**	**Median (IQR)**	***p*** **-value**	**Effect size** ^*^ **(95%CI)**	***p*** **-value** ^†^	***p*** **-value** ^†^	***p*** **-value** ^†^
						**Baseline vs. neurotablet**	**Neurotablet vs. standard Care**	**Baseline vs. standard care**
**Primary outcomes**								
Digit span forward	0.33 (0.33)	0.50 (0.12)	0.50 (0.17)	**<**0.001	0.45 (0.27 to 0.67)	**<**0.001	0.02	**<** 0.01
Digit span backward	4.00 (1.00)	4.00 (0.00)	4.00 (1.00)	0.04	0.13 (0.03 to 0.37)	0.10	0.66	0.14
Word list immediate	0.37 (0.15)	0.55 (0.17)	0.48 (0.28)	**<**0.001	0.52 (0.29 to 0.76)	**<**0.001	0.08	**<**0.01
Word list recall	0.33 (0.20)	0.40 (0.40)	0.40 (0.40)	0.35	0.04 (0.003 to 0.28)	0.53	0.99	0.53
Stroop—time	23.50 (17.50)	23.00 (13.50)	28.00 (27.50)	0.17	0.047 (0.01 to 0.30)	0.35	0.15	0.49
Stroop—errors	2.00 (7.00)	2.00 (3.00)	1.00 (5.00)	0.47	0.03. (0.003 to 0.23)	0.38	0.38	0.65
TMT-A	59.00 (45.00)	50.00 (50.00)	66.00 (83.00)	**<**0.01	0.20 (0.03 to 0.52)	0.11	0.02	0.04
TMT-B	235.00 (378.00)	200.00 (203.00)	272.00 (350.00)	0.11	0.09 (0.01 to 0.32)	0.26	0.65	0.06
TMT B-A	174.00 (121.00)	150.00 (117.00)	185.00 (118.00)	0.72	0.01 (0.002 to 0.24)	0.86	0.86	0.86
Complex figure recall	0.26 (0.24)	0.50 (0.25)	0.40 (0.41)	**<**0.01	0.22 (0.09 to 0.45)	**<**0.001	0.22	0.09
Phonemic fluency test	34.00 (15.00)	37.00 (15.00)	30.00 (16.00)	**<**0.01	0.25 (0.05 to 0.57)	0.05	**<**0.01	0.02
**Secondary outcomes**								
MoCA	22.00 (6.00)	23.00 (5.00)	22.00 (4.00)	0.92	0.003 (0.001 to 0.16)	0.90	0.90	0.90
CDT	12.00 (5.00)	12.00 (7.00)	13.00 (7.00)	0.76	0.01 (0.001 to 0.17)	0.57	0.57	0.57
Complex figure copy	0.84 (0.28)	0.80 (0.20)	0.70 (0.30)	0.32	0.05 (0.003 to 0.26)	0.52	0.49	0.49
Category fluency test	15.00 (6.00)	11.00 (5.00)	12.00 (3.00)	0.04	0.13 (0.03 to 0.35)	0.10	0.75	0.04
Naming	1.00 (0.07)	1.00 (0.00)	1.00 (0.00)	**<**0.01	0.25 (0.12 to 0.46)	0.02	0.36	0.10

^*^Kendall's W value.

^†^Benjamini-Hochberg correction.

MoCA, Montreal Cognitive Assessment; CDT, Clock Drawing Test; TMT-A, Trail Making Test A; TMT-B, Trail Making Test B; TMT B-A, Trail Making Test B-A.

Values are highlighted in bold and underlined if they are below the significance threshold (*p* < 0.05).

**Table 2:** Linear mixed-effects (LMM) model results for each primary and secondary outcome.

	**Fixed effects**		**Random effects**	
**Variables**	**Coefficients**	***p*** **–values**	**Subject SD**	**Residual SD**
**Primary outcomes**				
Digit span forward			0.08	0.11
Neurotablet	0.18	**<**0.001		
Standard care	0.10	**<**0.01		
Digit span backward			0.66	0.57
Neurotablet	0.36	0.03		
Standard care	0.08	0.62		
Word list immediate			0.11	0.09
Neurotablet	0.16	**<**0.001		
Standard care	0.11	**<**0.001		
Word list recall			0.20	0.14
Neurotablet	0.04	0.35		
Standard care	0.05	0.25		
Stroop—Time			24.47	41.83
Neurotablet	−10.82	0.36		
Standard care	4.12	0.73		
Stroop—errors			4.44	4.41
Neurotablet	−1.02	0.42		
Standard care	0.78	0.53		
TMT-A			45.13	48.92
Neurotablet	−9.96	0.48		
Standard care	24.88	0.08		
TMT-B			142.46	79.02
Neurotablet	−26.28	0.25		
Standard care	17.88	0.43		
TMT B-A			57.31	38.00
Neurotablet	1.80	0.87		
Standard care	1.28	0.91		
Complex figure recall			0.16	0.17
Neurotablet	0.18	**<**0.001		
Standard care	0.12	0.01		
Phonemic fluency test			9.11	4.31
Neurotablet	2.40	0.05		
Standard care	−3.32	**<**0.01		
**Secondary outcomes**				
MoCA			3.52	2.51
Neurotablet	−0.08	0.91		
Standard care	−0.60	0.40		
CDT			3.49	2.39
Neurotablet	−0.48	0.48		
Standard care	−0.72	0.29		
Complex figure copy			0.17	0.16
Neurotablet	0.01	0.85		
Standard care	−0.05	0.31		
Category fluency test			2.27	3.06
Neurotablet	−1.84	0.04		
Standard care	−1.84	0.04		
Naming			0.04	0.06
Neurotablet	0.05	**<**0.01		
Standard care	0.03	0.08		

Fixed and random effects are reported.

Values are highlighted in bold and underlined if they are below the significance threshold (*p* < 0.05).

### Primary outcomes

The Friedman's test analysis revealed that, between the three assessments, individuals with PD showed significant differences in TMT-A (*p* < 0.01, ES: 0.20, 95%CI 0.03 to 0.52), Phonemic fluency (*p* < 0.01, ES: 0.25, 95%CI 0.05 to 0.57), Digit Span Forward (*p* < 0.001, ES: 0.45, 95%CI 0.27 to 0.67), Digit Span Backward (*p* = 0.04, ES: 0.13, 95%CI 0.03 to 0.37), Complex Figure Recall (*p* < 0.01, ES: 0.22, 95%CI 0.09 to 0.45), and Word List Immediate (*p* < 0.001, ES: 0.52, 95%CI 0.29 to 0.76).

After Benjamini-Hochberg correction, significant differences between Baseline and Neurotablet for Word List Immediate (*p* < 0.001), Digit Span Forward (*p* < 0.001), Phonemic fluency test (*p* = 0.05) and Complex Figure Recall (*p* < 0.001) were found. Improved performance was demonstrated after the experimental training, compared to Baseline. Benjamini-Hochberg correction also showed significant differences between Neurotablet and Standard Care for TMT-A (*p* = 0.02), Phonemic fluency (*p* < 0.01) and Digit Span Forward (*p* = 0.02). Finally, Benjamini-Hochberg correction showed significant differences between Baseline and Standard Care for TMT-A (*p* = 0.04), Digit Span Forward (*p* < 0.01), Word List Immediate (*p* < 0.01) and Phonemic fluency test (*p* = 0.02).

The LMM revealed significant effects of conditions on Digit Span Forward (Neurotablet: beta = 0.18, *p* < 0.001; Standard care: beta = 0.10, *p* < 0.01), Digit Span Backward (Neurotablet: beta = 0.36, *p* = 0.03), Word List Immediate (Neurotablet: beta = 0.16, *p* < 0.001; Standard care: beta = 0.11, *p* < 0.001), Complex Figure Recall (Neurotablet: beta = 0.18, *p* < 0.001; Standard care: beta = 0.12, *p* = 0.01), and Phonemic fluency test (Neurotablet: beta = 2.40, *p* = 0.05; Standard care: beta = −3.32, *p* < 0.01). Except for Digit Span Forward and Complex Figure Recall, variables' variability was attributable to between-participants differences.

### Secondary outcomes

The Friedman's test analysis revealed that at the three assessments, individuals with PD showed significant differences in Category fluency test (*p* = 0.04, ES: 0.13, 95%CI 0.03 to 0.35) and Naming (*p* < 0.01, ES: 0.25, 95%CI 0.12 to 0.46).

After Benjamini-Hochberg correction, significant differences between Baseline and Neurotablet for Naming (*p* = 0.02) were found, which demonstrates improved performance after the experimental training, compared to the Baseline. Concerning Neurotablet vs. Standard Care, we did not find any significant differences in other cognitive scores. A significant difference was found in the Category fluency test (*p* = 0.04) between Baseline and Standard Care.

The LMM revealed significant effects of Conditions on Naming (Neurotablet: beta = 0.05, *p* < 0.01), and Category fluency test (Neurotablet: beta = −1.84, *p* = 0.04; Standard care: beta = −1.84, *p* = 0.04). In both cases, unexplained within-individual variability over time is slightly larger than the variability attributable to differences between individuals.

## Discussion

In recent years, the necessity to ensure the continuity of care at home has led to an increased emphasis on telemedicine and its potential applications in the field of neurorehabilitation. The present pilot randomized cross-over study was designed to evaluate the effectiveness of a new HB-CCT program delivered by the Neurotablet platform with respect to Standard Care in individuals with PD.

### Primary outcomes

This study demonstrated the positive effects of the HB-CCT in individuals with PD, indicating an enhancement in specific cognitive abilities. A statistically significant difference was observed between the HB-CCT Neurotablet intervention vs. Standard Care in three cognitive domains: verbal short-term memory, attentive capacities, and executive function skills. Specifically, we found an improvement in the primary outcomes Digit Span Forward, TMT-A, and Phonemic fluency tests. Few previous studies demonstrated the effect of HB-CCT programs in individuals with PD prioritizing the training of specific abilities, such as working memory (Edwards et al., 2013; Fellman et al., 2020; Ophey et al., 2020). Edwards et al. (2013) found an improvement of processing speed showing significant differences between post-training and Baseline, likewise our findings showed significant differences in processing speed between HB-CCT Neurotablet and Standard care. These studies suggest that the greater the degree of focus on a specific cognitive domain in training, the greater the likelihood of achieving improvements in that domain (Gavelin et al., 2022).

Overall, the effects of CCT on cognitive functioning have been demonstrated in several studies involving individuals with PD. París et al. (2011) evaluated the efficacy of a CCT on verbal short-term memory in individuals with PD, underlining a statistically significant enhancement in this ability following the completion of 12 forty-five-minutes supervised training sessions. Promising results have also been reported for subjects with MCI who received the intervention individually at home. For example, Bahar-Fuchs et al. (2017) utilized the CogniFit software that works in an individually-tailored and adaptive way, similarly to the Neurotablet software. In line with our findings, the authors found improvements in composite measures of memory (including verbal short-term memory) immediately post-training, as well as at three-month follow-up. Moreover, a single-blinded, randomized control pilot study on community-dwelling MCI patients analyzed the effects of a HB-CCT (Baik et al., 2024). It consisted of three times (around 24 min) a week sessions for 8 weeks, and it was implemented with the software Neuro-World, which trained several cognitive functions including attention, visual perception, memory, and executive functions. In alignment with the findings of our study, the post-training intervention demonstrated a notable enhancement in verbal short-term memory abilities when compared to the Baseline. We may conclude that the administration of CCT, whether in laboratory or home settings, may exert a beneficial impact on verbal short-term memory in patients with different neurodegenerative conditions.

We did not find significant differences in set-shifting neither between HB-CCT Neurotablet intervention and Standard Care, nor between HB-CCT Neurotablet intervention and Baseline. In line with our findings, previous studies investigating the effects of CCT on these domains in individuals with PD revealed no significant differences with the Baseline (Naismith et al., 2013; Ophey et al., 2020). In other studies, set shifting ability in trained individuals with PD was found to benefit from training also when compared to control participants (París et al., 2011; Alloni et al., 2018; Bernini et al., 2021). Given the difference in settings, one might speculate that performance in set shifting tests may be sensitive to the experimental setting (Guglietti et al., 2021). A controlled setting may facilitate patients in focusing on the task at hand, thereby enhancing the efficacy of the training programme.

Bernini et al. (2021) enrolled a group of PD-MCI patients who were trained with the CoRe software These findings may support the hypothesis that attention and executive functions are the primary cognitive abilities affected in individuals with PD, which may slow down the efficacy of general domain training on various cognitive tasks in a laboratory setting. Similar to our findings, the authors did not obtain a significant result for working memory in the post-training period when compared to the Baseline. Ophey et al. (2020) employed a targeted training programme on working memory in a cohort of non-demented PD patients and observed no significant differences in working memory outcomes, either post-training or in comparison with the control group. One possible explanation is that the training was too challenging, exceeding the cognitive resources available, and therefore preventing successful performance immediately post-training.

Furthermore, when looking at the HB-CCT Neurotablet in comparison to Standard Care, a positive impact was observed with regard to Phonemic fluency. The existing literature on the effects of CCT on word production in individuals with PD has yielded inconclusive results (Alloni et al., 2018; De Luca et al., 2019; Bernini et al., 2021). Our findings suggest that Phonemic fluency is susceptible to the intervention as individuals with PD demonstrated better performances after the HB-CCT, when compared to Standard Care. Attention and executive functions are the primary cognitive abilities affected in individuals with PD, which may slow down the efficacy of general domain training (Wallace et al., 2022). Nevertheless, when patients are exposed to a training targeting cognition, improvements in their executive abilities may be observed. Interestingly, Phonemic fluency performance declined significantly following the administration of Standard Care, when compared to the Baseline outcome.

Concerning the comparison between HB-CCT Neurotablet intervention vs. Baseline in the primary outcomes, we found an improvement in executive functions, learning ability, verbal short-term memory and visual long-term memory, namely in the following measures: Phonemic fluency test, Word List Immediate, Digit Span Forward and Complex Figure Recall.

In line with our findings, several studies have also found significant improvements in the post-training period as compared to the Baseline period in executive tests and learning trials that involved the presentation of verbal cues to memorize (Baik et al., 2024; Naismith et al., 2013; Petrelli et al., 2014). Instead, following the completion of the experimental intervention, we observed no significant result in long-term memory assessed by verbal tasks, in comparison to the Baseline condition. Literature showed positive effects of CCT on verbal long-term memory only when compared to the Baseline, and not in comparison to other training (París et al., 2011; Petrelli et al., 2014). The discrepancy with the existing literature may be explained by the type of exercises implemented in Neurotablet. The training included a greater number of visual and verbal items to be recalled immediately than long-term memory exercises. Therefore, the type of exercises and of stimuli implemented in the training may influence the corresponding cognitive domain assessed in post-training. Moreover, consistently with our results, previous studies have identified post-training improvement with respect to the control condition in visual long-term memory, although none of these were HB-CCT (París et al., 2011; Alloni et al., 2018).

The present study revealed no significant difference in performance on the Digit Span Backward task (measure of working memory) between HB-CCT Neurotablet intervention vs. Baseline. Conversely, Fellman et al. (2020) demonstrated that PD patients who underwent a 5-week training programme (comprising three 30-min sessions per week) exhibited a notable enhancement in working memory abilities, both in response to treated and untreated stimuli. One possible explanation for these results may stem from the emphasis of the training on practicing a specific cognitive domain.

Finally, when looking at the Standard Care vs. Baseline conditions we found worse performances in learning ability and executive functions, particularly in the following measures: Word List Immediate, Phonemic fluency, and TMT-A tests. Furthermore, we identified a reduction in verbal short-term memory as assessed by the Digit Span Forward. The implementation of an intervention that does not target cognitive abilities does not result in enhanced cognitive performance. These findings suggest that such an approach may not mitigate the neurodegenerative process. Consequently, there is a clear necessity for cognitive training to foster cognitive skills in PD.

### Secondary outcomes

The analysis did not reveal significant differences in any of the secondary outcomes between HB-CCT Neurotablet intervention and Standard Care. However, when comparing HB-CCT Neurotablet intervention vs. Baseline, some significant results emerged. Interestingly, the participation in the Neurotablet training increased picture naming performance in individuals with PD. Neuropsychological testings pre- and post- CCT typically did not include the assessment of linguistic abilities. Thus far, only one study investigating the effects of a HB-CCT in PD used the Boston Naming Test to assess language (Ophey et al., 2020). The authors revealed no improvement at the test after the training. However, a training specifically targeting working memory was used, differently from our study where a multiple domain training was included. It is noteworthy that few studies involved language exercises in the training (Clare et al., 2003; Gavelin et al., 2020). The improvement we found after Baseline may be attributed to the introduction of language training. Consequently, it may be valuable to incorporate such training into future CCT studies involving individuals with PD, given that cognitive impairments in this population frequently manifest in linguistic domains (Palmirotta et al., 2024).

A notable finding was that the analysis of global cognitive outcomes after the experimental training did not yield any statistically significant results when compared with Standard Care and with Baseline. A recent meta-analysis by Gavelin et al. (2022) empathized the effects of CCT on global cognitive efficiency, differently from prior meta-analyses (Leung et al., 2015; Orgeta et al., 2020). Multiple domain programs are more likely to be successful for global cognitive outcomes compared to programs targeting a single cognitive domain, whose effects tend to be most pronounced in the specific domains they target (Gavelin et al., 2022). The meta-analysis included both HB-CCT and CCT studies, using as global cognitive outcomes MoCA or MMSE. We may speculate that our results differ from those exposed by Gavelin et al. (2022) because they did not distinguish HB-CCT from laboratory based CCT. The setting of administration of the training has an impact on global performances (Guglietti et al., 2021). Also, it is well established that distinct measurements for cognitive changes show different sensitivity in detecting cognitive decline (Biundo et al., 2016). The methodological differences between our study and existing literature make direct comparison of results challenging. As the number of HB-CCT studies on PD increases in future, it will become possible to make informed speculation about the effects of training on global efficiency.

In terms of the secondary outcomes, there was no evidence that visual spatial and constructive abilities benefited from the Neurotablet training. This finding is in line with previous literature (Ophey et al., 2020). Furthermore, a systematic review and meta-analysis revealed that visuo-constructive abilities demonstrated the least benefit from CCT across all cognitive domains (Sanchez-Luengos et al., 2021).

The implementation of a multiple domain training programme, conducted in group sessions, yielded favorable outcomes in terms of Category fluency (París et al., 2011). Nevertheless, our study did not reveal any significant improvement in this ability following the experimental training, whether in comparison with the Baseline or with the post Standard Care. Similarly, Alloni et al. (2018) obtained comparable outcomes when conducting CCT individually. It may be the case that the administration of training in groups could prove beneficial with regard to cognitive improvement. Factors such as the capacity to directly supervise participants in order to guarantee adherence and compliance, to furnish motivational support and encouragement, and to resolve IT issues as they arise, in addition to augmented social interaction for participants, are indispensable to enhance cognitive abilities, particularly Category fluency (Guglietti et al., 2021). Finally, a comparison between the Baseline and Standard Care groups revealed a decline in Category fluency. This may be attributed to the neurodegenerative process that is ongoing and not amenable to intervention through Standard Care.

### Limitations and future directions

This study is not without limitations. A fundamental limitation of the cross-over study is the potential for carryover effects to obscure the impact of the training. In future studies, it would be advisable to incorporate a washout period between the two interventions, which could be useful to mitigate the risk of the carryover effect. Importantly, our findings cannot be generalized to items related to daily life. It would be crucial to explore the effects of the HB-CCT training on every-day activities. Our study did not measure functional outcomes such as quality of life and activities of daily living. Therefore, future studies will need to use these measures to understand how the cognitive domains trained in the exercises can benefit patients' daily lives. Furthermore, a limit of our findings is the lack of follow-up measures. Further research may address generalization and aftereffects of adaptive HB-CCT in a PD population. Another limitation of our study is the small sample size, as the present work is a pilot study, therefore these results need to be confirmed in larger samples. A potential consequence of the small sample size is low statistical power, which can have several implications for the reliability of results, specifically in detecting subtle cognitive effects after the intervention. For example, low power increases the risk of Type II errors leading the researchers to conclude there is no effect when, in reality, some exist.

Among the participant inclusion criteria, we adopted the MoCA cut-offs reported in Santangelo et al. (2015). While these thresholds may seem lower than some widely accepted standards, they were selected based on their application within a similar demographic context. We acknowledge the potential risk of including participants with more severe cognitive impairments, particularly given our sample's higher educational levels. Future research could benefit from exploring a broader array of normative data across different cultural contexts to validate these thresholds further and enhance the generalizability of our findings.

In this pilot study, we did not assess the participant's technological literacy, a potential predictor of intervention adherence. However, we did not register dropouts related to technology issues, which indicates that the participants did not have specific difficulties using the tablet. It may be possible that a cohort of older adults may be less familiar with tablets and technological platforms than the general population, however, in a previous study, we demonstrated that older adults find it easier to use tablets compared to computers than younger individuals (Canini et al., 2014). Further research may be conducted to examine the evolution of these technologies, with the aim of developing new, tailored telerehabilitation solutions that address any significant challenges encountered with existing devices and platforms.

Finally, in future it will be useful to implement an active control group, such as engaging participants in non-adaptive or placebo cognitive tasks, to better isolate the intervention's unique effects and avoid the risk that increased cognitive engagement could explain the results rather than the specific benefits of HB-CCT.

## Conclusions

This pilot study demonstrates that HB-CCT using Neurotablet may be an effective method for improving specific cognitive abilities in PD patients, including short-term verbal memory, long-term visual memory, and phonemic fluency, as compared to the standard care. Improvements were observed in targeted areas, though results for other cognitive functions, such as processing speed and set-shifting, were mixed, with no significant differences between groups. These findings underscore the potential of HB-CCT as an innovative, accessible cognitive training tool suitable for home use, providing essential support in managing cognitive symptoms in neurodegenerative diseases.

## Data Availability

The raw data supporting the conclusions of this article will be made available by the authors, without undue reservation.
