# Supplementary material for: Effectiveness of a home-based computerized cognitive training in Parkinson's disease: a pilot randomized cross-over study
Source: Front Psychol. 2025 Jan 9;15:1531688. doi: 10.3389/fpsyg.2024.1531688 (PMC11754226; doi:10.3389/fpsyg.2024.1531688)
Supplement: Supplementary file 1 [file Data_Sheet_1.pdf]

Supplementary Materials

Material and Methods

Experimental Intervention (Neurotablet®)

The Neurotablet contains a series of exercises aimed at training different cognitive functions. In order to adapt the tasks to the unique peculiarities of each patient, each exercise provides the option of modifying the parameters.
The proposed exercises are presented in the following table.

Table S1. Exercises implemented in the Neurotablet cognitive training.

Attention	Memory	Neglect	Executive Functions	Language
Auditory and visual alerting	Spatial memory span	Description of figures	Color Stroop	Anagrams
Acoustic and visual selective attention	Memory Pathways	Copying of matrices	Motion Stroop	Association sound symbol
Alternate acoustic and visual attention	Memory N back	Path reproduction	Flow Free	Spelling judgments
Acoustic visual divided attention	N back self-determined			Visual comprehension
Modality-specific and crossmodal training				Pattern copying
				Dictation

Figure S1. Illustrative examples of the exercises implemented in the Neurotablet cognitive training.

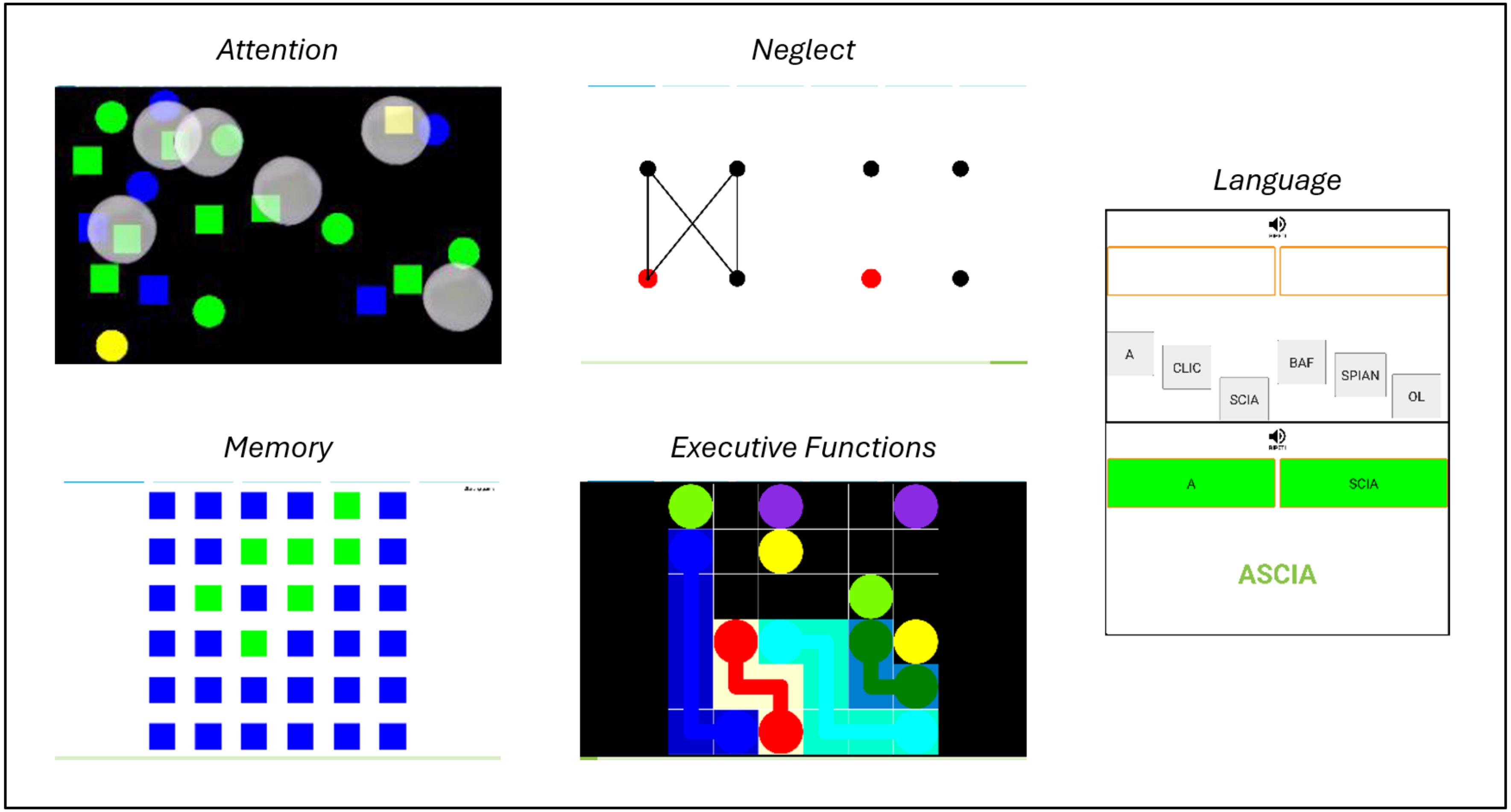

Figure S2. Training platform illustration on tablet devices.

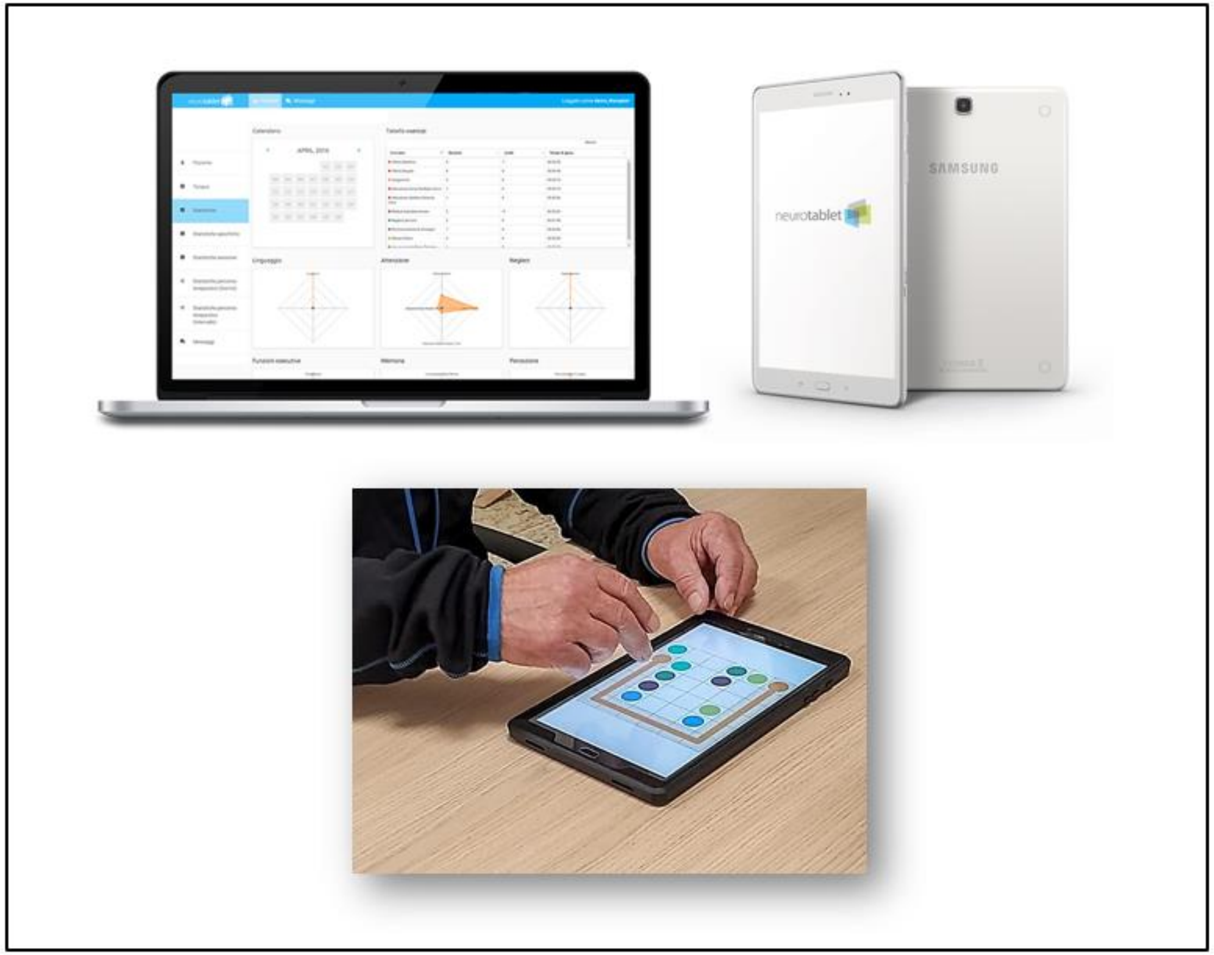

Table S1. Descriptive analysis of demographic and clinical data in the PD population.

Variables	Mean ± SD
Age [yrs]	69.32 ± 7.21
Sex (%)	
Male	18 (72.00)
Female	7 (28.00)
Education [yrs]	13.00 ± 4.51
Disease duration [yrs]	9.04 ± 8.12
LEDD	561.92 ± 267.71
H&Y	2.52 ± 1.04
MDS-UPDRS III	33.76 ± 14.25

LEDD: levodopa (l-dopa) equivalent daily dosage; H&Y: Hoehn and Yahr Scale; MDS-UPDRS III: MDS-Unified Parkinson's Disease Rating Scale.

Table S2. Neuropsychological tests used during the cognitive assessments.

**Neuropsychological Tests**	**Description**	**Main Outcomes**
Montreal Cognitive Assessment (MoCA) and parallel italian versions	MoCA is a screening test consisting of 12 subtests analyzing: (1) memory (delayed recall of five nouns); (2) visuo-spatial abilities (a clock-drawing task and copy of a cube); (3) executive functions (a brief version of the Trail Making B, a phonemic fluency task, and a two-item verbal abstraction task); (4) attention, concentration and working memory (target detection using tapping; a serial subtraction task, and forward and backward digit span); (5) language (a naming task with animals, repetition of two sentences); (6) temporal and spatial orientation (current year, month, exact date, and day of the week; name of this place, and which city it is in).	Number of correct trials.
Rey Auditory Verbal Learning Test (RAVLT)	RAVLT is a test of learnin and verbal episodic memory. RAVLT Immediate test is structured of 5 consecutive repetitions of 15 unrelated words that the subject is invited to learn. A free recall 30 minutes later occurs (RAVLT Recall).	Number of words correctly remembered.
Rey-Osterrieth Complex Figure (ROCF) Recall	ROCF - Recall investigates visual long-term memory. Participants were asked to draw a figure as best they could recall, approximately 10 minutes after having copied and memorized the complex figure.	Number of units recalled and drawn, according to accuracy and placement of units.
Digit Span Forward and Backward	Digit Span is a test of short-term and working memory. The examiner presents orally a series of random numbers generally at one digit per second. After each sequence has finished, in the Forwards version the participant is required to repeat the same digits exactly as presented, while in the Backwards version, the participant has to repeat the same digits in the reverse order.	Number of correct trials in the forward condition and backward condition, respectively.
Trail Making Test (TMT A-B)	TMT A-B is a measure of basic attention and working memory. The first part (A) assesses psychomotor processing speed and visual scanning. An array of numbers on a page is shown to the subjects and they are instructed to draw lines connecting the numbers in sequential order within the	Time (seconds) to completion of each trial.

	allowed time. The second part (B) provides cognitive flexibility features: psychomotor processing speed, visual scanning, and attentional set-shifting. An array of numbers and letters are shown to the subjects and they are asked to draw connecting lines while alternating between numbers and letters in sequential order.	
Stroop test - Brief version	Stroop is a measure of set shifting and attention performed in 3 conditions: 1) black words appear; 2) different colored circles appear (blue, green, red); 3) colored words appear (blue, green, red). In condition 1, the participant is instructed to read as quickly as possible the correct black words and the correct colored circles. In condition 3 (Inhibition), the color words are written in a different color with respect to their real one (i.e., blue is printed in red). The participant has to name the color of the ink in which the word is printed.	Time (seconds) to completion of each trial, the difference in color-naming speed, and the number of errors in the color-word interference effect.
Phonemic and Category fluency test	Two tests of verbal production and executive functions, the participant has to say as many words as possible during 1 minute, starting with a specific letter (Phonemic Fluency) or belonging to a specific category (Category Fluency).	Number of words produced over all trials.
Clock Drawing Test	The participant is presented with a blank circle and asked to fill in the numbers of a clock face and set hands on a specific time (e.g., 6:05).	Number of errors in the sequence and presence of the numbers within the clock, and the presence and placement of the clock’s hands.
Rey–Osterrieth Complex Figure (ROCF) Copy	ROCF - Copy investigates visuo-spatial constructional functions and planning. Participants have to copy the ROCF on a sheet. ROCF - Copy includes 18 units and the maximum score is 36.	Number of units drawn, according to accuracy and placement of units.
Naming of the Screening for Aphasia in NeuroDegeneration (SAND)	The participant is asked to name 14 black and white images within 5 seconds.	Number of correct trials.
Repeatable Battery for the Assessment of	RBANS is a battery consisting of 12 subtests, subdivided according to cognitive domains.	The main outcomes for subtests.

Neuropsychological Status (RBANS) - parallel versions A and B	**Immediate Memory**<br><br>- *List Learning*: immediate recall of 10 unrelated words after four learning trials.<br>- *Story Memory*: immediate recall of a story after two trials of listening.<br><br>**Visuo-spatial/Constructional**<br><br>- *Figure Copy*: copying a geometric figure formed by 10 units.<br>- *Line Orientation*: the participant has to identify the correct matching 10-item line and a radiating array of 13 lines spanning 180 degrees.<br><br>**Language**<br><br>- *Picture Naming*: consists of naming 10 pictures individually presented.<br>- *Semantic Fluency*: the participant has to say as many words as possible during 1 minute belonging to a specific category.<br><br>**Attention**<br><br>- *Digit Span*: the examiner reads a number of sequences of increasing length and asks participants to repeat them in the same order.<br>- *Coding*: the participant has to write in the boxes the number corresponding to that of the symbol in the string.<br><br>**Delayed Memory**<br><br>- *List Recall*: involves the free recall of the words from the List Learning.	**Immediate Memory**<br><br>- *List Learning*: number of words correctly remembered.<br>- *Story Memory*: number of items of the story correctly remembered.<br><br>**Visuo-spatial/Constructional**<br><br>- *Figure Copy*: number of units drawn, according to accuracy and placement of units.<br>- *Line Orientation*: number of correctly matched lines: number of items completed in 90 seconds.<br><br>**Language**<br><br>- *Picture Naming*: number of correct trials.<br>- *Semantic Fluency*: number of words produced over all trials.<br><br>**Attention**<br><br>- *Digit Span*: number of correct trials<br>- *Coding*:<br><br>**Delayed Memory**<br><br>- *List Recall*: number of correctly free recalled words of the list.<br>- *List Recognition*: number of words correctly recognised.

	- *List Recognition*: involves recognition testing for memory of the words from the List Learning.<br>- *Story Recall*: involves free recall of the story from the Story Memory.<br>- *Figure Recall*: involves free recall of the figure from the Figure Copy.	- *Story Recall*: number of correctly free recalled items of the story.<br>- *Figure Recall*: number of units recalled and drawn, according to accuracy and placement of units.

Results

Table S3. Mann-Whitney test analysis of demographic and clinical data in the PD population at baseline.

	Group 1	Group 2	
**Variables**	**Mean ± SD**	**Mean ± SD**	**p-value**
Age [yrs]	67.53 ± 7.26	72 ± 6.58	0.33
Education [yrs]	13.53 ± 5.06	12.20 ± 4.39	0.20
H&Y	2.37 ± 1.09	3.15 ± 1.35	0.30
MoCA	21.20 ± 3.45	22.30 ± 3.18	0.42

H&Y: Hoehn and Yahr Scale; MoCA: Montreal Cognitive Assessment.
